# Real-Time Monitoring of Photoinduced pH Jumps by *In Situ* Rapid-Scan EPR Spectroscopy

**DOI:** 10.1021/acs.jpclett.4c00564

**Published:** 2024-07-01

**Authors:** Florian Johannsen, Lara Williams, Man Him Chak, Malte Drescher

**Affiliations:** † Department of Chemistry and Konstanz Research School Chemical Biology, University of Konstanz, Universitätstraße 10, 78464 Konstanz, Germany; ‡ School of Chemistry, UNSW Sydney, Sydney, NSW 2052, Australia

## Abstract

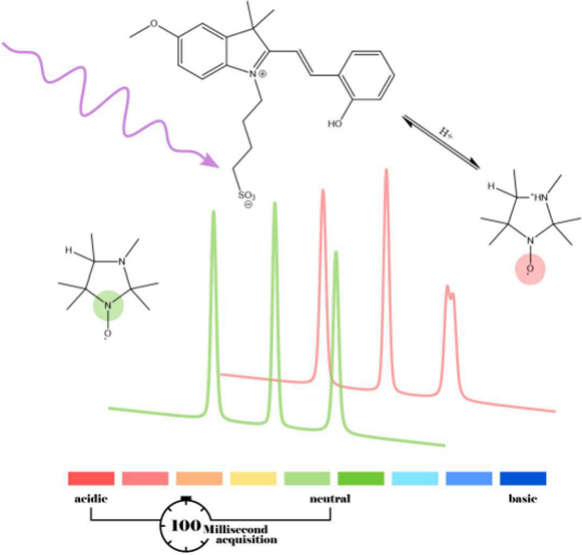

This work represents the first demonstration of monitoring kinetics upon a light-induced pH jump by *in situ* rapid-scan (RS) electron paramagnetic resonance (EPR) spectroscopy on the millisecond time scale. Here, we focus on the protonation state of an imidazolidine type radical as a pH sensor under visible light irradiation of a merocyanine photoacid in bulk solution. The results highlight the utility of photoacids in combination with pH-sensitive spin probes as an effective tool for the real-time investigation of biochemical mechanisms regulated by changes in the pH value.

One of the key physiological parameters in life science is the pH value. The pH regulates enzyme activity and transport of substances, ensures the stability of biomolecules, and significantly influences the folding and functionality of proteins. Typical spectroscopic methods employed for sensing pH-dependent mechanisms on the subsecond time scale are fluorescence spectroscopy,^1^ Fourier-transform infrared spectroscopy,^2^ or time-resolved nuclear magnetic resonance (NMR) spectroscopy.^3^ Electron paramagnetic resonance (EPR) spectroscopy, however, provides complementary insights into biological systems and their interactions that are not readily accessible through other spectroscopic methods. A fundamental problem of using EPR for real-time pH determination is the restricted excitation bandwidth of pulsed measurements and the limited time resolution of traditional continuous wave (CW) experiments. Acquisition of a CW EPR spectrum often takes several minutes but can also take longer depending on the system under study, the spin concentration, or the required signal-to-noise ratio. Rapid-scan (RS) EPR, on the other hand, substantially improves the signal-to-noise ratio and time resolution. More specifically, it allows much higher microwave powers to be applied before saturating the spin system while being largely unaffected by bandwidth related constraints.^4^ Rapid-scan is thus well suited to resolve spectral changes of the EPR resonance line, accompanied by switching the pH with photoacids. Photoacids are molecules that release protons upon illumination, lowering the pH value of aqueous solutions.^5^ Light as an external trigger offers properties that are important in performing successful time-resolved RS experiments for biological studies. Light can be used with high spatial and temporal control, is not harmful to proteins at wavelengths above 300 nm, and unlike titration (with acids and bases) does not alter the sample volume or resonator tuning.

A metastable photoacid that switches with visible light is protonated merocyanine. Merocyanine and its derivatives are among the most widely studied photoacids, allowing reversible and pronounced pH jumps from basic to acidic environments. They exhibit fast response times with proton transfer reactions on the subsecond time scale, making them ideal for studies of fast biochemical dynamics.^6^ Monitoring of the pH jump upon proton release is accomplished by the addition of pH-sensitive nitroxides. In particular, nitroxides of the imidazolidine type have been extensively studied for pH determination.^7^ Imidazolidine nitroxides are stable,^8^ both chemically and thermally, highly sensitive, and cover an exceptionally wide pH range.^9^

Taken together, photoacid, pH-sensitive nitroxide, and rapid-scan EPR spectroscopy form an excellent toolbox for studying various biochemical processes regulated by changes of the pH value.

This paper describes the most important characteristics of the pH-sensitive nitroxide and photoacid used in this work and provides details about the experimental setup, the synthesis of the required chemicals, and data processing. We present first measurements of *in situ* RS-EPR pH monitoring and demonstrate how the pH of the solution can be controlled by changes of the sample composition or laser emission. The structure of the imidazolidine type nitroxide used for pH monitoring in this study is shown in Figure 1a. A special property in contrast to its common form is the presence of a photoprotecting group, which is meant for future studies, e.g., experiments on samples containing further paramagnetic species. The functionality of the nitroxide itself is well understood,^10^ and we will therefore only briefly discuss its most important characteristics. Imidazolidine type nitroxides have a second nitrogen atom that can be protonated depending on the pH of the solution. Protonation of the nitrogen at lower pH leads to a shift of the spin density toward the oxygen atom of the NO group which consequently lowers the isotropic hyperfine splitting (Figure 1c). Hence, these nitroxides have two spectroscopically distinguishable protonated and unprotonated forms. Differences between the two species, in terms of hyperfine splittings and g-factors, can be best seen in the high-field line. Spectra at intermediate pH values are comprised of a superposition of the acidic and basic form of the nitroxide (Figure 1d) with splittings well described by the Henderson–Hasselbalch equation
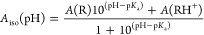
1where *A*(R) and *A*(RH^+^) are the isotropic hyperfine coupling constants for the nonprotonated basic and protonated acidic form of the nitroxide. The isotropic hyperfine splitting determined for the nitroxide used in this work is approximately 1.3 G, making the spin probe suitable as a pH indicator over the range of about three pH units centered at a p*K*_a_ of 4.7. Figure 1b depicts the structure of the merocyanine photoacid (MCH) in its protonated form. MCH is a metastable photoacid with exceptional bulk pH switching properties allowing large tunable, reversible, but long-lived pH jumps up to 3.5 units.^11^ Activation of the photoacid (MCH) leads to a sudden proton release and subsequent ionization of the pH-sensitive nitroxide. CW EPR experiments allow determination of the shift in the isotropic hyperfine splitting at constant pH values (Figure 1c), but they cannot cover the overall process of ionization in a time-resolved manner. The recorded titration curve, however, can be used as a calibration for time-resolved rapid-scan measurements. This allows the pH value to be determined from the splitting of the nitroxide peaks in reverse. For all measurements conducted, we aimed to induce the pH jump right at the turning point of the titration curve, where sensitivity is highest. Protonation of the nitroxide is well resolved by acquiring RS spectra with a 1 s time resolution (Figure 2a). The excellent time resolution of RS EPR, however, allows us to capture the process of ionization also on the millisecond time scale (Figure 2b). The light-induced proton transfer reaction to the nitroxide radicals is clearly evident. Spectra measured with 100 ms time resolution allow accurate determination of the point in time where the protonated and deprotonated forms are present at equal concentrations. This is readily apparent from the high-field peak that after the onset of laser irradiation separates into two distinct slopes (Figure 2c) revealing the precise hyperfine splitting of the two forms of the radical. The distances between the central peak of the triplet and the two observable slopes agree very well with the two outermost splittings of the CW titration curve. The corresponding pH values are read out from the splitting between the low- and center-field peaks (Figure 2d).

**Figure 1:**
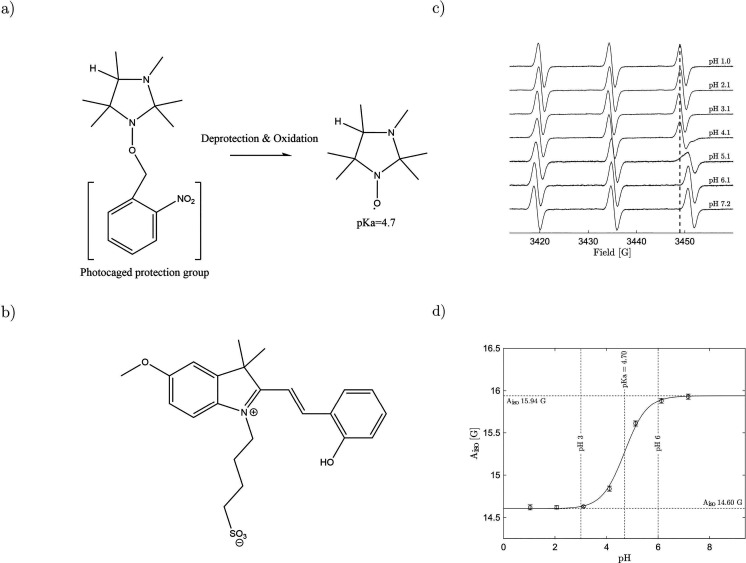
(a) pH-sensitive nitroxide before and after cleavage of the photoprotecting group. (b) Photoacid. (c) Titration experiment: CW EPR spectra were taken at different pH values. Top to bottom: pH 1.0, 2.1, 3.1, 4.1, 5.1, 6.1, 7.2. (d) pH dependence of the isotropic hyperfine splitting measured as the distance between the low- and central-field components (circles). Fit to the Henderson–Hasselbalch titration curve (solid line).

**Figure 2:**
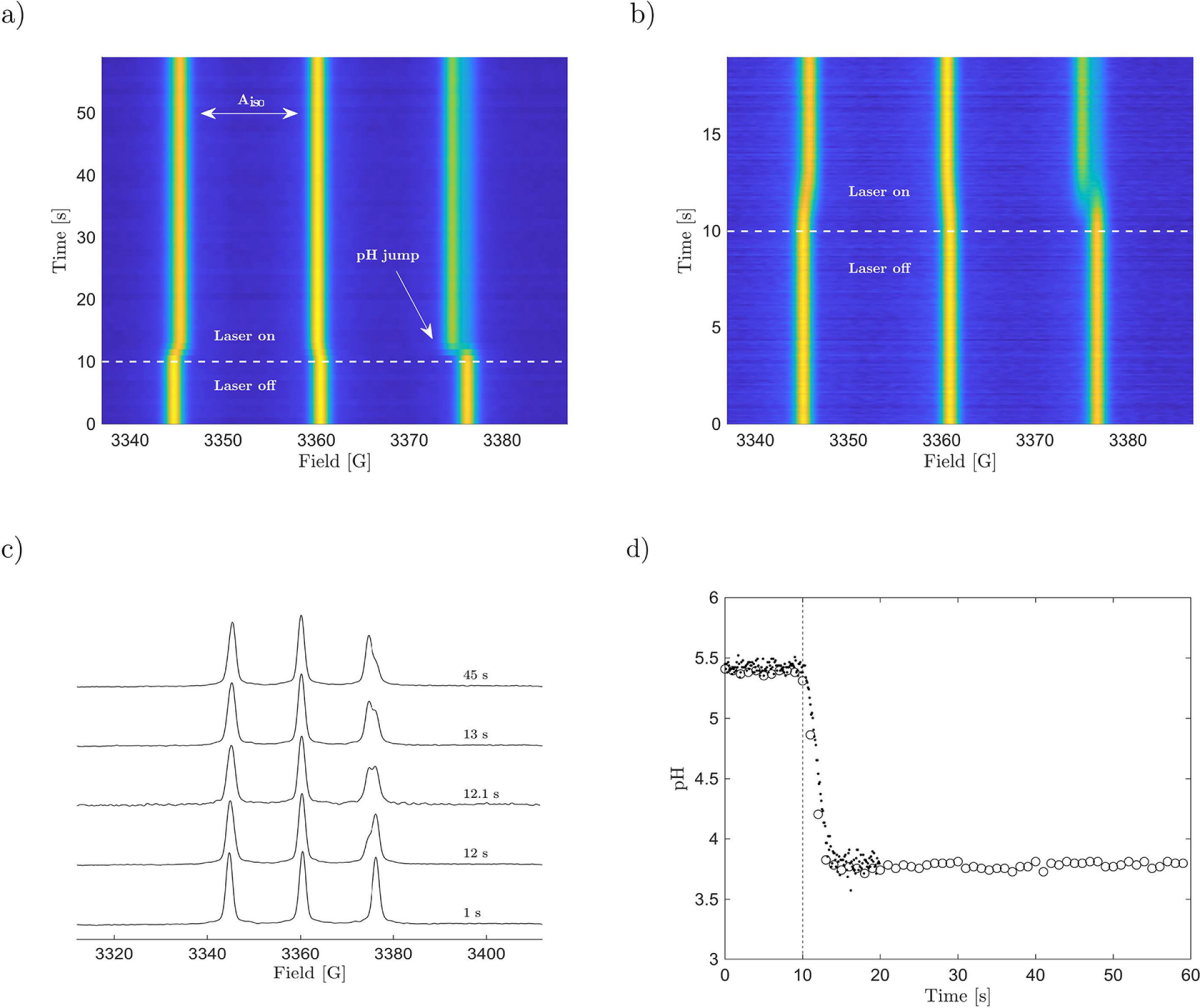
Time-resolved rapid-scan experiment. (a) pH jump and corresponding decrease in the hyperfine splitting of the pH-sensitive nitroxide radical. Signal intensities are color-coded (view from above). A 100 G sinusoidal scan modulated at 10 kHz is used to acquire spectra with (a) 1 s or (b) 100 ms time resolution. The laser was switched on after 10 s of measuring time (450 nm, 3 mJ). (c) Slices along the magnetic field axis. Bottom to top: 1, 12, 12.1, 13, 45 s. (d) pH readout from the spectra in (a) circles and from the data shown in (b) dots.

We now demonstrate that the pH jump is reversible with thermal recovery on the time scale of minutes and that the solution’s pH can be maintained under constant irradiation. Precise regulation of the proton transfer reaction is accomplished by either the sample’s composition, the energy, or the wavelength of the laser. Activation of the photoacid followed by thermal recovery is depicted in Figure 3a. Switching off the laser after a short-term exposure of the sample results in the photoacid slowly returning to its stable isomer. This process is coupled with a gradual increase in the hyperfine splitting. In contrast, splitting of the triplet remains constant under continuous irradiation (Figure 3b); that is, the solution’s pH stays unchanged. At the beginning of the photoinduced reaction, there is a rapid increase in proton concentration as MCH dissociates quickly under light exposure. As time progresses, the capacity of protonated molecules depletes, and the solution reaches a new equilibrium. The time evolution of the isotropic hyperfine splitting (or pH-Jump respectively) is well captured by a logistic decay.
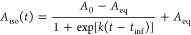
2*A*_0_ and *A*_eq_ are the isotropic hyperfine splittings at zero time and at equilibrium. *k* denotes the decay constant, and *t*_inf_ represents the time at which the function reaches half of its maximum value, i.e., the inflection point. Lowering the energy of the laser visibly decelerates the pH jump but does not delay the onset of proton release. Reducing the concentration of the photoacid ultimately leads to fewer protons being released into the solution, i.e., a smaller pH jump. Another useful way to gain control of photoinduced pH jumps is varying the excitation wavelength around the absorption maximum of the photoacid (here, 435 nm). The energy and wavelength of the laser as well as the sample’s composition therefore allow the pH value of the solution to be precisely controlled (Figure 3c). A more comprehensive overview of the experiments conducted and the parameters determining the dynamics of photoinduced pH jumps is shown in Figure 4 and Table 1.

**Figure 3:**
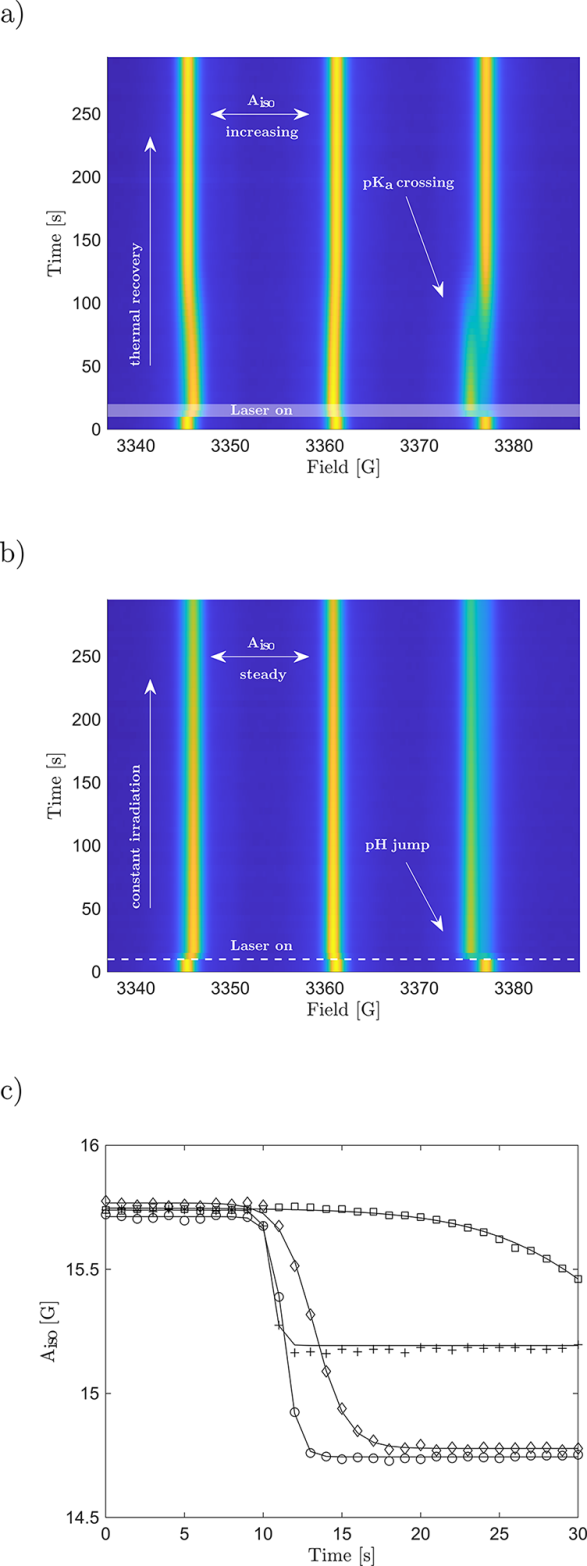
(a) pH jump and thermal recovery. Laser irradiation (450 nm, 3 mJ) is indicated by a transparent horizontal. (b) Constant irradiation. (c) Influence of the sample composition and laser emission: 450 nm, 3 mJ, *c*(MCH) = 1500 μM (circles); 450 nm, 1 mJ, *c*(MCH) = 1500 μM (diamonds); 450 nm, 3 mJ, *c*(MCH) = 1125 μM (plus signs); 535 nm, 1 mJ, *c*(MCH) = 1500 μM (squares). All samples were irradiated after 10 s of measuring time.

**Table 1:** pH-Jump Experiments and Determination of Kinetic Parameters

pH_initial_	pH_final_	ΔpH	*t*_inf_ [s]	δpH [s^–1^]	c(MCH) [μM]	λ [nm]	Pulse energy [mJ]
5.4	3.8	1.6	1.4	0.55	1500	450	3
5.5	3.8	1.7	1.8	0.51			2.5
5.4	3.7	1.7	2.3	0.42			2
5.5	3.9	1.6	2.4	0.37			1.5
5.5	3.9	1.6	3.2	0.31			1
						
5.5	3.8	1.7	8.5	0.21		525	1
5.5	3.9	1.6	23.7	0.07		535	
5.6	5.3	0.3	39.0	0.01		550	
							
5.5	4.6	0.9	0.5	0.37	1125	450	3
5.4	5.4	0	–	–	750		

**Figure 4:**
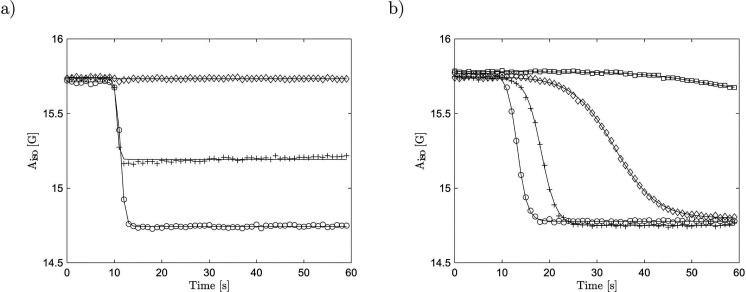
(a) Change of the isotropic hyperfine splitting depending on the photoacid concentration: 1500 μM (circles), 1125 μM (plus signs), 750 μM (diamonds). Samples were irradiated at 450 nm and a pulse energy of 3 mJ. (b) Change of the splitting depending on the wavelength of the laser: 450 nm (circles), 525 nm (plus signs), 535 nm (diamonds), 550 nm (squares). The pulse energy was kept at 1 mJ.

Photoacids offer spatial and temporal control of the pH, making them extremely attractive for noninvasive fast and well synchronized time-resolved biology studies. Activation of the photoacid accompanied by proton release and subsequent ionization of the pH-sensitive nitroxide can be monitored in real time by *in situ* RS EPR spectroscopy. The pH of the solution can be determined without delay from the hyperfine splitting of the nitroxide. Constant laser irradiation maintains the pH of the solution with the possibility to precisely control its value by adjusting the laser emission or photoacid concentration. The kinetics studied here are well captured with a time resolution of seconds. RS EPR, however, offers the opportunity of recording the proton transfer reaction on the millisecond time scale. The available time resolution will surely prove crucial for investigating biochemical processes through photoinduced pH jumps in the near future. It is also worth mentioning that imidazolidine-type nitroxides exhibit tunable p*K*_a_ values covering a wide pH range by selection of various substituents on the nitroxide moiety. Notably, there also exist metastable photobases that function similarly to photoacids: they donate OH^–^ ions and thus increase the solution’s pH upon irradiation.^12^ In conclusion, the experiments presented open up entirely new perspectives to use spin probes for kinetic measurements. This work represents a novel area of research that offers great potential for studying biological systems with RS EPR spectroscopy.

## Materials and Methods

MCH was synthesized as previously described.^11^ The pH-sensitive nitroxide comprising a photocaged protecting group was purchased from Enamine Ltd. Cleavage of the ortho-nitrobenzyl group with subsequent oxidation of the EPR silent hydroxylamine to the desired radical is achieved after 2 min of irradiation under a 8 W UV hand lamp at 302 nm. CW EPR titration experiments (Figure 1) were carried out on an EMXnano benchtop X-Band spectrometer (Bruker) using a set of standard buffer solutions purchased from ROTICalipure. Samples contained 19 μL of the buffer solution and 1 μL of the pH-sensitive nitroxide taken from a 10 mM stock solution in DMSO. Five CW EPR X-Band spectra were collected for each pH value with a modulation amplitude of 1 G, a microwave power of 1 mW, 100 G sweep width, and 120 s acquisition time. Averaging of the obtained hyperfine splitting values is used to minimize deviations in the sample composition during titration. Rapid-scan experiments were conducted with the Bruker rapid-scan accessory on an Elexsys spectrometer at X-Band, ambient temperatures, and a microwave power of 20 mW. The cavity of the resonator (Bruker Biospin ER4125RS) operates in the TE_011_ mode with a center frequency of 9.43 GHz. A 100 G sinusoidal scan modulated with 10 kHz is used to acquire the transient spin response with 100 ms, 1 s, or 5 s time resolution. Unless otherwise stated, all samples contained 15.5 μL of Milli-Q-water, 1.5 μL of the nitroxide, and 3 μL of the photoacid. Nitroxide and photoacid were dissolved in a 10 mM DMSO stock solution. Two minutes of deprotection resulted in an approximate spin concentration of 100 μM. Activation of the photoacid is carried out with a diode pumped Nd:YAG laser (EKSPLA) operating at a fixed repetition rate of 50 Hz and a pulse length of 4 ns. All samples were irradiated after 10 s of measuring time with the optical fiber directly coupled into the resonator from above. Control experiments (data not shown) with TEMPO (a commonly used nitroxide that lacks an additional nitrogen atom which can be protonated) did not reveal any changes of the resonator Q-factor or coupling upon irradiation. The achievable time resolution is scan-rate-dependent and determined by the sweep time (100 μs at 10 kHz modulation frequency, 50 μs at 20 kHz etc.) and the number of scans (a minimum of 16 is required by Xepr). There is no time delay between the individual spectra. Spectrometer and lasers are independent systems that are operated separately. As a consequence, a single spectrum may be acquired partly before and partly after switching on the laser. Spectra are background corrected with the Xepr-software provided by Bruker. Data postprocessing further included the combination of both half-cycles of the rapid-scan period and the Kramers–Kronig relation to add the corresponding absorption spectra calculated from the in-phase component of the incident microwave.^13^ Deconvolution is used to preserve accurate lineshapes, although no passage effects were observed.^14^ The signals are finally smoothed by convolution with a Gaussian function whose full width at half-maximum was set to 0.5 G.

## Appendix

Dynamics of the isotropic hyperfine splitting (or pH jump respectively) can be modeled by a logistic decay. Table 1 provides an overview to specify such measurements. Given are the initial and final pH-value; the total change ΔpH after 60 s of measurement time; the inflection point *t*_inf_, at which the pH jump is half completed (relative to the time of laser irradiation); as well as the gradient δpH at this point.
